# Successful Closed Reduction of a Lateral Elbow Dislocation

**DOI:** 10.1155/2016/5934281

**Published:** 2016-12-21

**Authors:** Kenya Watanabe, Takuma Fukuzawa, Katsuhiro Mitsui

**Affiliations:** Department of Orthopedics, Nagano Prefectural Suzaka Hospital, Suzaka, Nagano, Japan

## Abstract

In this report, we present a case of lateral elbow dislocation treated with closed reduction. Lateral elbow dislocation is rare, and a closed reduction is reported with even less frequency. The reduction can be hindered by swelling and soft tissue interposition, and we describe the use of a nonoperative reduction technique performed under mild sedation with early physiotherapy to avoid joint stiffness. No additional complication was observed, and the normal range of elbow movement and function was obtained by early physiotherapy.

## 1. Introduction

Elbow dislocation is a common injury, and posterior dislocation, specifically, is a frequent form. Simple lateral elbow dislocation is rare and often associated with neurovascular issues with difficulty in performing closed reduction [1]. Reduction can be hindered by swelling and soft tissue interposition. In this rare case of lateral dislocation treated with closed reduction under mild sedation, we describe the use of a nonoperative reduction technique and early physiotherapy to avoid joint stiffness.

## 2. Case Report

**Figure 1:**
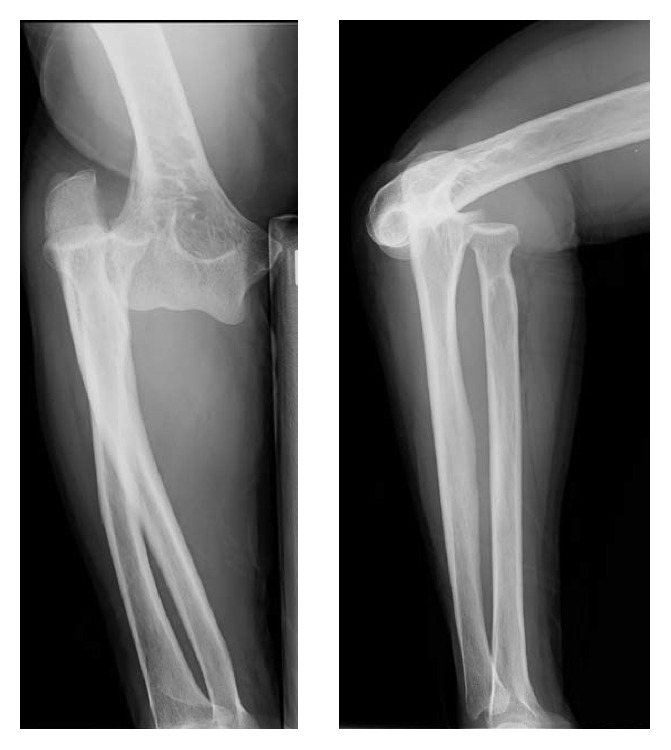
Anteroposterior and lateral radiographs of the elbow revealed lateral convergent displacement of the radius and ulna relative to the humerus without fracture signs.

A 68-year-old woman fell on her right side with her right elbow flexed while carrying her luggage. After the trauma, she found her elbow in a valgus position and repositioned the joint herself through flexed and internal rotation. She was admitted to the emergency center of our hospital because of right elbow pain and deformity. She presented with a flexed elbow and the hand placed on her abdomen. The neurologic examination revealed mild numbness in her 4-5th digits only. The radial pulse was palpable. Anteroposterior and lateral radiographs of the elbow showed lateral convergent displacement of the radius and ulna relative to the humerus without fracture signs (Figure 1).

Upon diagnosis of lateral elbow dislocation, we attempted closed reduction under sedation with 15 mg pentazocine hydrochloride and 25 mg hydroxyzine hydrochloride i.m. as well as local anesthesia with 10 mg lidocaine.

**Figure 2:**
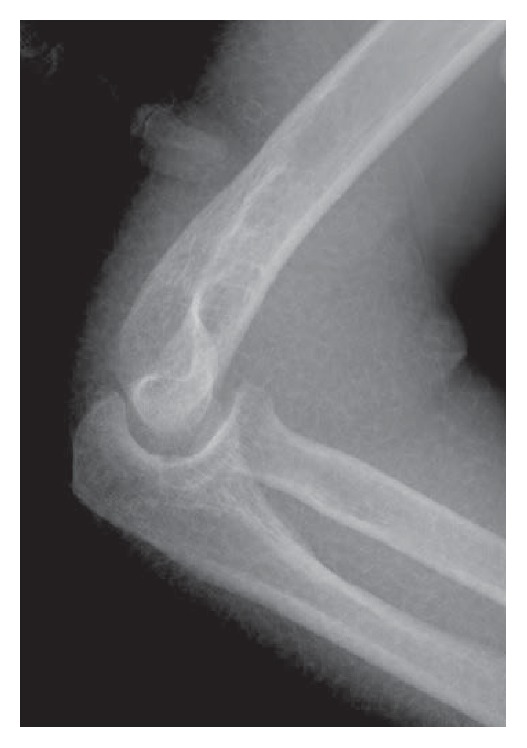
During reduction maneuver, the image intensifier revealed that only the ulnohumeral joint was reduced.

**Figure 3:**
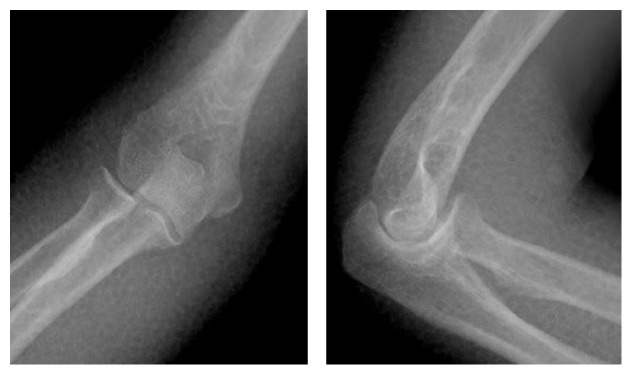
Postreduction radiographs revealed no fractures.

**Figure 4:**
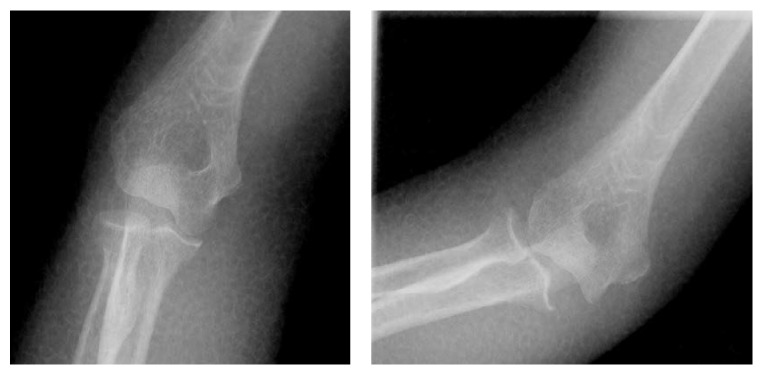
Postreduction radiographs revealed obvious varus and valgus instability of the joint. However, redislocation was not observed in the range of motion from 30° to 90°.

**Figure 5:**
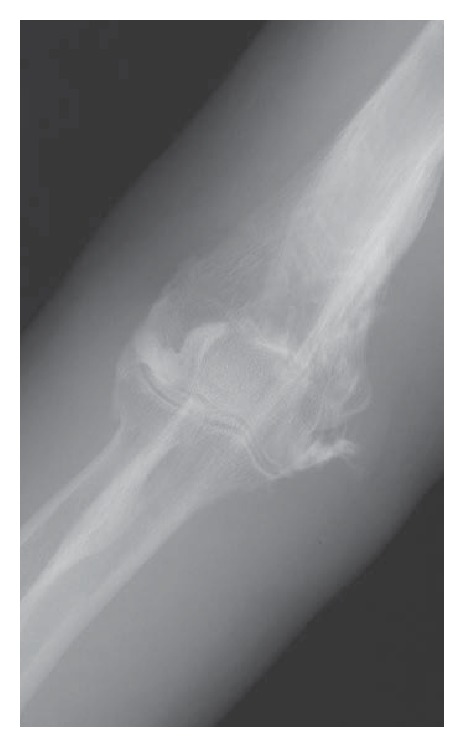
The elbow arthrography revealed a complete tear of the medial collateral ligament and a suspected partial tear of the lateral collateral ligament.

The reduction maneuver was performed in the image intensifier room with the patient in the supine position. Initially, we attempted gentle longitudinal traction on the axis in the flexed position with an assistant holding the axilla as a countertraction while simultaneously checking lateral images of the elbow. After the first try was unsuccessful, we applied longitudinal traction on the axis in the stretched position while checking anteroposterior images of the elbow. The operator distracted her wrist distally and moved the dislocated proximal forearm medially. After this maneuver, we felt a click; however, the image intensifier revealed that the elbow joint was not reduced fully, and we suspected that only the ulnohumeral joint was reduced on the trochlea of the humerus (Figure 2). The operator gently pushed the radial head with the elbow flexed and we heard a click to indicate full reduction. Postreduction radiographs were obtained without signs of fracture (Figure 3). Though obvious varus and valgus instability of the joint was observed, redislocation was not observed in the range of motion from 30° to 90° (Figure 4). Elbow arthrography revealed a completely torn medial collateral ligament (MCL) and a suspected partial tear of the lateral collateral ligament (LCL, Figure 5). CT scan and MRI revealed no sign of fracture and additional information. A motor nerve conduction velocity study of the ulnar nerve revealed decreased velocity and prolonged latency.

**Figure 6:**
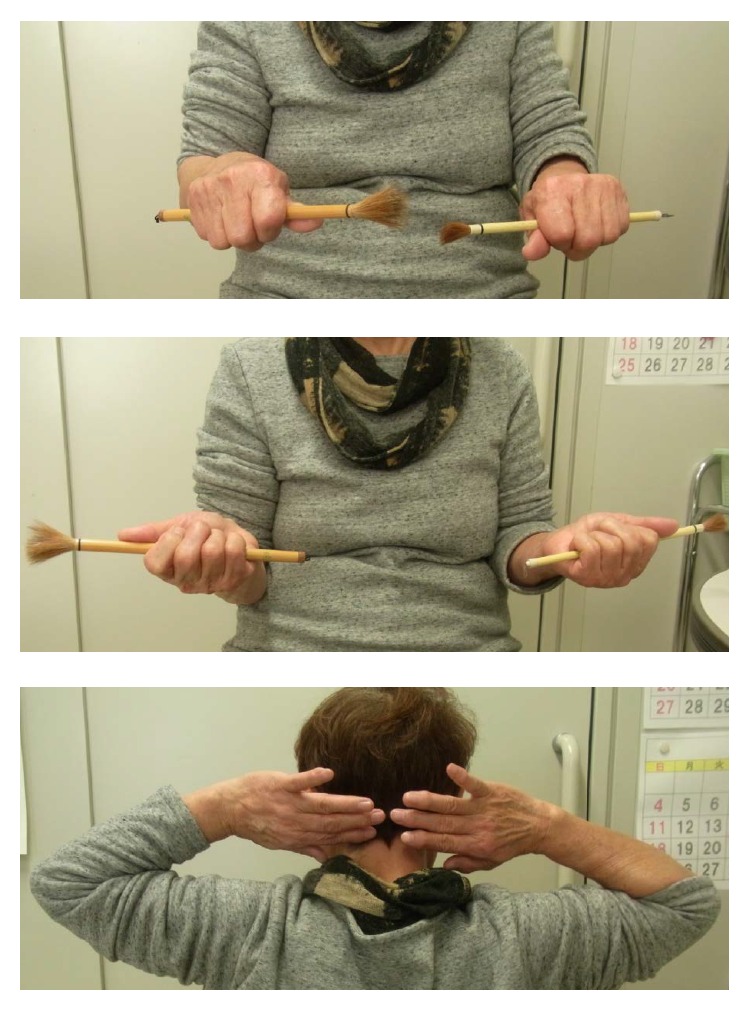
After 8 weeks, the elbow range of motion was from 0° to 125°, supination to 90°, and pronation to 90°.

**Figure 7:**
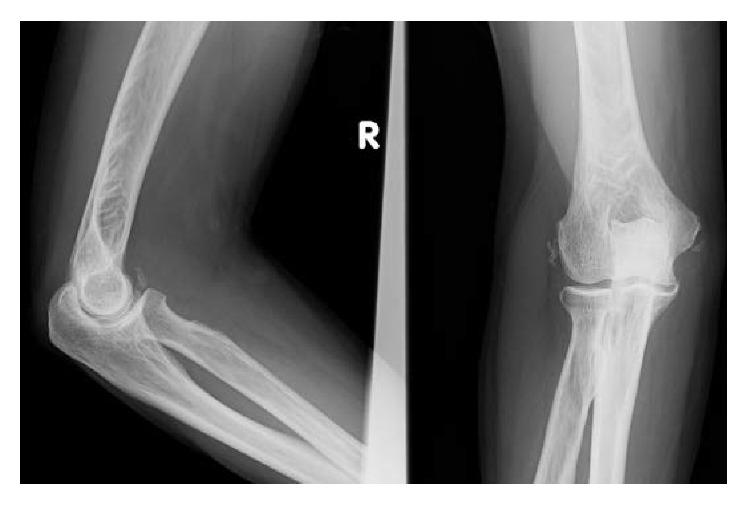
Eight weeks after the trauma, radiographs revealed mild calcification around the anterior articular capsule, medial collateral ligament, and lateral collateral ligament.

**Figure 8:**
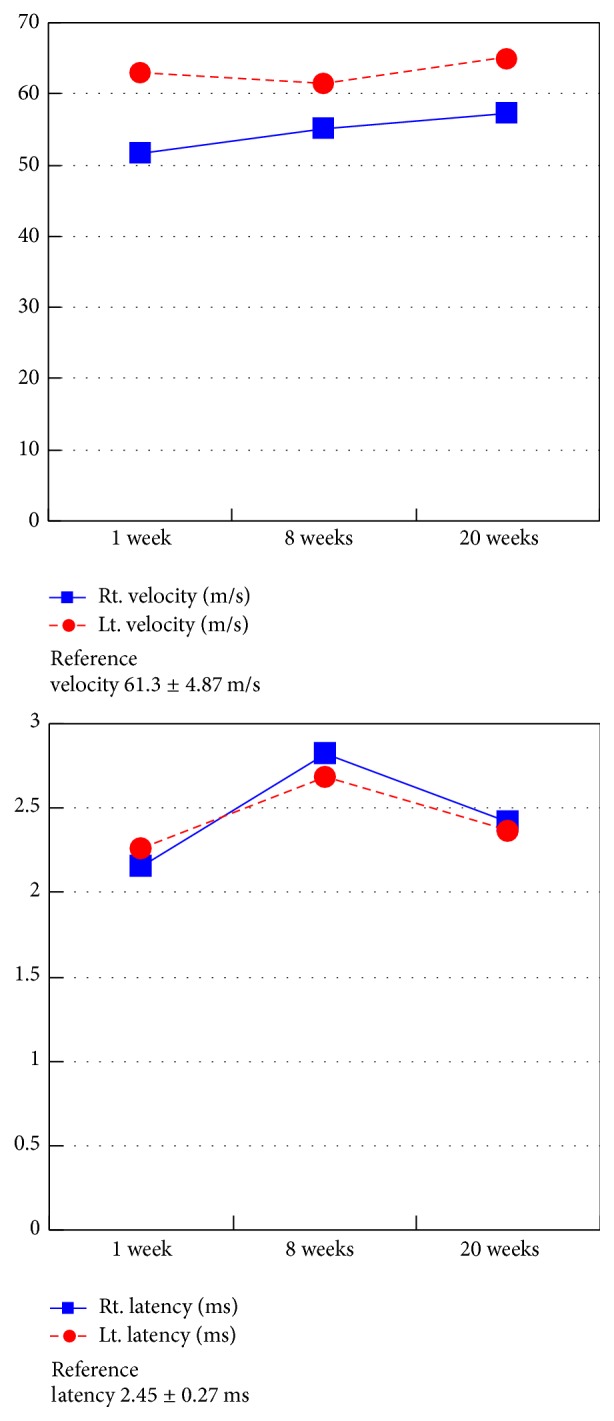
Decreased velocity and prolonged latency of the ulnar nerve improved steadily.

The elbow joint was immobilized at 90° of flexion with the forearm in supination in a posterior plaster cast for 3 weeks. Mild physiotherapy started at 2 weeks after the trauma and the elbow was examined weekly. The plaster was removed 3 weeks after the trauma. After 8 weeks, the elbow range of motion was from 0° to 125°, supination to 90°, and pronation to 90° (Figure 6). Radiographs revealed mild calcification around the anterior articular capsule, MCL, and LCL; however, the patient did not complain of elbow pain (Figure 7). The decreased velocity and prolonged latency of the ulnar nerve improved steadily (Figure 8). Medical examination 20 weeks after trauma measured her grip strength at 17 (right)/19 (left) kg, and she had not experienced any trouble in daily living. At 6 months, she had no sensory disturbance distributed by the ulnar nerve, and her score of the DASH -JSSH: Japanese Society for Surgery of the Hand version of the Disability of Arm, Shoulder, and Hand questionnaire [1] was 0.86.

## 3. Discussion

In a systematic review of the literature, three cases of lateral dislocation were reported in 342 patients with dislocated elbows, and one case of anterolateral dislocation was reported [2]. Ulnar nerve involvement after lateral dislocation is typically reported [3] as observed in our case. The reduction of pure lateral elbow dislocation is known to be difficult owing to high risk of incarceration such as swelling, soft tissue interposition such as anconeus muscle, brachialis muscle, ulnar nerve, or associated fractures [4, 5].

Several reduction maneuvers for pure lateral traumatic dislocation of the elbow have been reported. Modification of the gravity-aided “hanging arm” technique originally described for shoulder dislocations by Stimson (modified Stimson's technique [3]) and longitudinal traction on the axis in the semiflexed position, without forcing the elbow in extension [6], is reported as a reducing maneuver. In this case, reduction verified by the image intensifier suggests that this is a reliable method.

Even if obvious varus or valgus instability of the joint was observed, redislocation was not observed in flexion and extension indicating that simple lateral dislocation of the elbow joint can be treated without surgical treatment [3, 4, 6]. Physiotherapy started 2-3 weeks after trauma allowing patients to regain the normal range of elbow movements. Prolonged immobilization of the elbow joint after elbow dislocation is not recommended, as immobilization for more than 14 days may be associated with stiffness [2].

## 4. Conclusion

Lateral dislocation of the elbow joint is rare and its closed reduction is even rarer. Testing the stability is important for early physiotherapy to avoid joint stiffness. Excellent functional outcomes can be achieved even if followed by nonoperative therapy.

## References

[1] Imaeda T., Toh S., Nakao Y. (2005). Validation of the japanese society for surgery of the handversion of the disability of the arm, shoulder, and hand questionnaire. *Journal of Orthopaedic Science*.

[2] de Haan J., Schep N. W. L., Tuinebreijer W. E., Patka P., den Hartog D. (2010). Simple elbow dislocations: a systematic review of the literature. *Archives of Orthopaedic and Trauma Surgery*.

[3] Khan S. K., Chopra R., Chakravarty D. (2008). Successful closed manipulation of a pure lateral traumatic dislocation of the elbow joint using a modified Stimson's technique: a case report. *Journal of Medical Case Reports*.

[4] Exarchou E. J. (1977). Lateral dislocation of the elbow. *Acta Orthopaedica*.

[5] Chhaparwal M., Aroojis A., Divekar M., Kulkarni S., Vaidya S. V. (1997). Irreducible lateral dislocation of the elbow. *Journal of Postgraduate Medicine*.

[6] Gokcen B., Ozyurek S., Atik A., Sivrioglu A. K., Kaya E., Keklikci K. (2013). Successful closed manipulation of simple lateral dislocation of the elbow joint: a case report. *Oman Medical Journal*.

